# Theory-of-mind in individuals with Alström syndrome is related to executive functions, and verbal ability

**DOI:** 10.3389/fpsyg.2015.01426

**Published:** 2015-09-23

**Authors:** Hans-Erik Frölander, Claes Möller, Mary Rudner, Sushmit Mishra, Jan D. Marshall, Heather Piacentini, Björn Lyxell

**Affiliations:** ^1^Health Academy, School of Health and Medical Sciences, Örebro UniversityÖrebro, Sweden; ^2^Audiological Research Centre, Örebro University HospitalÖrebro, Sweden; ^3^Swedish Institute for Disability ResearchLinköping, Sweden; ^4^Linnaeus Centre HEADLinköping, Sweden; ^5^Research on Hearing and Deafness (HEAD) graduate SchoolLinköping, Sweden; ^6^Department of Audiology, Örebro University HospitalÖrebro, Sweden; ^7^Department of Behavioral Sciences and Learning, Linköping UniversityLinköping, Sweden; ^8^Institute of Health Sciences, Utkal UniversityBhubaneswar, India; ^9^Jackson LaboratoryBar Harbor, ME, USA; ^10^Alstrom Syndrome InternationalMount Desert, ME, USA

**Keywords:** Alström syndrome (AS), ciliopathy, deafblindness, theory-of-mind, verbal ability, executive functions

## Abstract

**Objective:** This study focuses on cognitive prerequisites for the development of theory-of-mind (ToM), the ability to impute mental states to self and others in young adults with Alström syndrome (AS). AS is a rare and quite recently described recessively inherited ciliopathic disorder which causes progressive sensorineural hearing loss and juvenile blindness, as well as many other organ dysfunctions. Two cognitive abilities were considered; Phonological working memory (WM) and executive functions (EF), both of importance in speech development.

**Methods**: Ten individuals (18–37 years) diagnosed with AS, and 20 individuals with no known impairment matched for age, gender, and educational level participated. Sensory functions were measured. Information about motor functions and communicative skills was obtained from responses to a questionnaire. ToM was assessed using Happés strange stories, verbal ability by a vocabulary test, phonological WM by means of an auditory presented non-word serial recall task and EF by tests of updating and inhibition.

**Results**: The AS group performed at a significantly lower level than the control group in both the ToM task and the EF tasks. A significant correlation was observed between recall of non-words and EF in the AS group. Updating, but not inhibition, correlated significantly with verbal ability, whereas both updating and inhibition were significantly related to the ability to initiate and sustain communication. Poorer performance in the ToM and EF tasks were related to language perseverance and motor mannerisms.

**Conclusion**: The AS group displayed a delayed ToM as well as reduced phonological WM, EF, and verbal ability. A significant association between ToM and EF, suggests a compensatory role of EF. This association may reflect the importance of EF to perceive and process input from the social environment when the social interaction is challenged by dual sensory loss. We argue that limitations in EF capacity in individuals with AS, to some extent, may be related to early blindness and progressive hearing loss, but maybe also to gene specific abnormalities.

## Introduction

The present study focuses on cognitive prerequisites for the development of theory-of-mind (ToM) in adolescents and young adults with Alström syndrome (AS). ToM refers to the ability to impute mental states to self and to others (Premack and Woodruff, 1978), of importance to establish social relations (Hughes and Leekam, 2004). A significant step in the development of this ability occurs during the preschool years around the age of four when children normally understand that another person may hold a belief different from themselves (Wellman et al., 2001). Cognitive skills such as the ability to process information and the ability to control one’s own thoughts and actions are important for the development of ToM (Wellman and Woolley, 1990; Pernier and Lang, 1999; Sabbagh et al., 2010). Deficiency in ToM is one of the core traits of Autism spectrum disorders (ASD; Baron-Cohen, 1989), but has also been observed in populations with other syndromes, including: Down syndrome (Zelazo et al., 1996); Fragile X syndrome (Belmonte and Bourgeron, 2006); Williams syndrome (Cornish et al., 2005) and CHARGE syndrome (Hartshorne et al., 2005). Clinical observations suggest that individuals with AS have a varying degree of ToM ranging from normal to levels typical of individuals with high functioning autism (Frölander et al., 2014).

Alström syndrome is an autosomal recessive syndrome within the Ciliopathy Spectrum. AS is rare but individuals identified with this syndrome are rapidly increasing (900, 2015 ISA). It is multi-systemic with high prevalence of additional diseases (Marshall et al., 2007a, 2011). AS causes progressive dual sensory loss, i.e., deafblindness (Möller, 2007). Sensorineural hearing loss progresses slowly in the first decade, usually reaching moderate or severe loss in the following decades. Age of onset, however, varies from infancy to adulthood. The high prevalence of otitis media in this group causes additional hearing loss. Cone rod retinal dystrophy leads to Retinitis Pigmentosa (RP) and juvenile blindness. Age of onset differs, but the onset of visual dysfunction is earlier than that of hearing loss, and typically established within weeks after birth (Marshall et al., 2007b). The visual loss deteriorates to blindness usually in late adolescence. (Marshall et al., 2011), and is, in contrast to hearing loss, already significant during the important stage of ToM development around the age of four. Motor milestones are often delayed in AS. Deficits in coordination, balance and fine motor skills have been observed, as well as early language delays and atypical behavior (Marshall et al., 2007a). The first and the second author have vast clinical experience of people with different deafblind syndromes, and the clinical findings in persons with AS includes lack of inhibition and as children extreme stubbornness and excessive eating (Frölander and Möller, 2015, Personal communication). Early hearing loss is associated with delayed development of ToM for children in hearing families who use speech communication, irrespective of the use of technical aids including hearing aids and cochlear implants (CI; Peterson, 2004). The importance of access to sound for the development of ToM in children who rely on speech communication has recently been shown in a study in which better ToM was demonstrated in congenitally deaf children who received CIs at an average of 18 months compared to those who received their implants at an average of 41 months (Sundqvist et al., 2014). It has thus been proposed that for children in hearing families, early hearing loss leads to impoverished social interaction, delaying the development of ToM. However, neither degree of hearing loss nor age at onset was found to be associated with ToM development in a population of individuals with AS. Access to sound would be expected to promote social interaction and thus development of ToM also in individuals with AS, but the generally slow progress of hearing loss might explain why no relationship between ToM and onset of hearing loss was observed in our previous study (Frölander et al., 2014). Studies of children with congenital blindness have demonstrated that a significant visual loss may also cause a delayed development of ToM (Minter et al., 1998; Roch-Levecq, 2006). In a previous study, age at onset of visual loss in AS was correlated with ToM, probably reflecting a loss of vision that is demonstrated within a few weeks from birth (Frölander et al., 2014). Such rapid vision loss is already evident by the sensitive age of four causing a lack of social and communicative stimuli with a negative impact on ToM development.

Performance on ToM tasks in typical individuals is related to working memory (WM). During ToM tasks information has to be kept in mind while determining states of mind. This loads on WM (Davis and Pratt, 1995; Hughes, 1998a,b; Keenan, 1998; Keenan et al., 1998). WM is an essential component in more complex cognitive activities such as communication (Baddeley, 2012). Previous results show that the AS group performs at a lower level in WM tasks compared to non-disabled controls. However, performance on WM tasks and ToM tasks was not significantly correlated in either group (Frölander et al., 2014). One reason for this might be that WM capacity beyond a critical level does not contribute to an enhanced performance (Slade and Ruffman, 2005).

Executive functions (EF) control and regulate thought and action (Espy et al., 2004; Burgess and Simons, 2005). EF include updating of new information, inhibition of irrelevant information and shifting of focus between different sources of information (Miyake et al., 2000; Letho et al., 2003). EF is closely associated with ToM in non-disabled populations (Hughes, 1998a; Perner and Lang, 1999; Mitchell and Riggs, 2000; Sabbagh et al., 2010; Zelazo and Carlson, 2012) as well as in disabled populations, including: ASD (Ozonoff and Jensen, 1999; Joseph and Tager-Flusberg, 2004) cerebral palsy (Li et al., 2007); frontal lobe damages (Rowe et al., 2001) and amygdala damage (Fine et al., 2001). Specifically, the role of inhibitory control has been stressed in the emergence and expression of ToM (Carlson and Moses, 2001; Carlson et al., 2002; Leslie et al., 2004). No previous study has examined the role of EF in ToM development in a population of individuals with AS.

Theory-of-mind is closely related to verbal ability irrespective of level of functioning (Slade and Ruffman, 2005), and this applies to individuals with AS (Frölander et al., 2014). Receptive language development is in general delayed in individuals with AS (Marshall et al., 2007b). EF is related to verbal ability in non-disabled populations (Carlson et al., 1998) as well as in individuals with ASD (Landa and Goldberg, 2005). EF is also important for communicative skills, such as ability to respond to conversational changes, in disabled as well as in non-disabled populations (Bishop and Adams, 1989; Hughes, 1998a; Ylvisaker and DeBonis, 2000).

In the present study we focus on how phonological WM capacity, executive functioning, verbal ability and communicative skills relate to development of ToM in a population of adolescents and young adults with AS, compared to a group of individuals with typical development matched for age and educational level. In addition, we examine how characteristics of sensory loss and motor deficits are related to development of ToM in AS.

We predict that verbal ability will be of particular importance for ToM performance in AS, as this relation has been displayed in a previous study, but also that EFs such as the ability to initiate and sustain communication will relate to ToM performance. We further assume that cognitive skills predict ToM performance in individuals with AS, in contrast to individuals with normal hearing and vision, by underpinning the ability to engage in communication.

## Materials and Methods

### Participants

Ten young adults (six females) with AS, and a mean age of 28.30 (6.08), participated in the study. It should be noted that seven out of ten individuals were the same individuals as reported in Frölander et al. (2014). Background information was obtained from medical records and from responses to the Alström Syndrome International (ASI) questionnaire by the participants and their families (Alström Syndrome International, 2010).

#### Hearing

All subjects showed a bilateral symmetrical moderate to profound sensorineural hearing loss. The onset of hearing loss was either congenital or early childhood. The hearing loss was in all subjects progressive but with variable rate. All subjects were at the time of the testing fitted with bilateral hearing aids. Hearing impairment (HI) was assessed by pure tone audiometry with calculation of the average pure tone threshold for the best ear at frequencies 0.5, 1, 2, and 4 KHz (PTA_4_). The audiograms were performed with standard equipment. The hearing tests used were either audiometry performed in standardized settings within 6 months and/or by audiometry performed at the time of testing by the use of audiometry with earphones (Kuduvawe Ltd).

#### Vision

Visual acuity was measured using Snellen chart-based standard tests and visual field was measured using Goldmann perimetry, resulting in categorization into five phenotypes; 1 = normal, 2 = presence of a partial or complete ring scotoma, the latter either extending or not extending into periphery, 3 = concentric central field loss with a remaining peripheral island less than one half of the field circumference, 5 = no visual field, blindness (Grover et al., 1997). All subjects were legally blind since childhood, and 7/10 had no residual vision at all (**Table 1**).

**Table 1:** Participant characteristics (mean + SD) for non-disabled individuals (*n* = 20), individuals with Alström syndrome (AS) (*n* = 10), individuals with AS and better Theory-of-mind (ToM) (*n* = 3), and individuals with AS and poorer ToM (*n* = 7).

	*n*	AS	*p*	AS better	AS poorer	*P*
Age	29.30 (4.69)	28.30 (6.08)	ns	32.67 (6.66)	27.00 (2.22)	ns
Gender	20 (12 females)	10 (6 females)	ns	3 (2 females)	7 (4 females)	ns
Educational level	3.30 (0.57)	3.10 (0.99)	ns			
Verbal ability	69.38 (8.23)	48.00 (21.03)	<0.01^∗∗^			
Verbal ability	69.38 (8.23)			62.00 (20.88)		ns
Verbal ability	69.38 (8.23)				41.50 (18.06)	<0.01^∗∗^
Visual acuity		0.032 (0.041)		0.039 (0.052)	0.029 (0.040)	ns
Visual field		4.75 (0.45)		4.33 (0.58)	4.89 (0.33)	ns
Hearing PTA_4_		63.18 (11.24)		61.67 (7.63)	63.75 (0.67)	ns

*^∗∗^Difference is significant at p < 0.01*.

Data about vestibular functioning was retrieved from medical records. Information about occurrence of repetitive mannerisms (i.e., hand or finger flapping or twisting or complex whole-body movements) and difficulties balancing were obtained from responses to a questionnaire (Alström Syndrome International, 2010). The information was categorized in five levels, due to reported frequency of occurrence, from “never” to “almost always.”

A control group of 20 non-disabled individuals (eight males) with normal hearing and vision were chosen to match the AS group with no differences regarding gender, age, and educational level (defined by years of schooling).

As a previous study revealed substantial variance in ToM performance in the AS group it was planned to divide the group into two sub groups enabling more specific comparisons with the control group regarding number of correct mental inferences produced (Frölander et al., 2014). It was thus decided that individuals with AS producing correct mental inferences within 1 SD of the mean of the control group (*n* = 3), would be compared separately with the control group, to determine if their performance in other domains resembled that of the control group. These individuals are referred to as the group of AS individuals displaying a better mental state understanding, equal to the control group. The remainder of the group of individuals with AS (*n* = 7) was also compared with the control group, to determine how their performance differed in other domains. This group is referred to as the group of AS individuals displaying a poorer mental state understanding than the control group. There was no difference between the two subgroups of AS individuals in gender frequency, age or educational level. In addition, no difference whether in hearing, visual acuity or visual field was found between the better and the poorer ToM – performing group.

The study was approved by the regional ethics review board of Uppsala – Örebro, Sweden and the institutional Review Board of The Jackson Laboratory, USA. Informed consent was obtained from all participants.

### Measures and Scoring

#### Advanced ToM

Theory-of-mind was measured by a collection of stories from Happe’s (1994) advanced test of ToM – the strange stories (Fletcher et al., 1995; Happe et al., 1996; Jolliffe and Baron-Cohen, 1999; White et al., 2009). The stimuli presented involved eight stories including two examples of each of double bluff, persuasion, white lies, and misunderstanding.

The stories were read aloud to the participants. The participants were offered the opportunity to read the stories in Braille or have one more verbal presentation.

A “why question,” concerning the ability to understand mental states was asked in connection with each of the stories (for example: Peter thinks his aunt looks silly in her new hat, but when she asks if he likes it, he answers that it is very nice, why?) The answer could be scored as correct or incorrect. The answer to the question could furthermore either be physical or mental. Physical state answers were associated with consequences for example “to get something.” Mental state answers referred to thoughts, feelings, desires, traits and dispositions including terms as “think,” “know” “like” and “lie.” A score of 2 was given if participants referred to exact mental states. 1 was given for correct mental state answers in more general terms, or for a correct physical state answer. 0 was given if the answer was unrelated. The score for the ToM stories ranged from 0 to 16 (Happe, 1994). Inter-rater reliability between one of the authors and an independent rater was 92%.

#### Phonological Working Memory

Phonological WM was assessed using Serial Recall of Non-words (Gathercole and Pickering, 2000), taken from a computer based test battery, the Sound Information Procesing System (i.e., the SIPS, Lyxell et al., 2009). Non-word series (for example: “med, tas, rog”) with increasing lengths, 3 – 5 words, were used as stimuli, in total 6 series. The prerecorded auditory signals were presented on a laptop, from the computer platform, in order to keep the time of the presentation of stimuli constant. The built in loudspeakers on the laptop computer were used for presentation. The participants were asked to repeat each of the non-words after each serie of non-words. The answers were recorded as a basis for later transcription and scoring of accuracy according to three different criteria: (1) The percentage of correctly reproduced non-words; (2) The percentage of correctly reproduced consonants; (3) The percentage of non-words pronounced with the correct vowel and total number of syllables.

#### Executive Functions

The EFs of updating and inhibition were measured using a shortened and adapted version of the Cognitive Spare Capacity Test (CSCT; Mishra et al., 2013b). The prerecorded auditory signals were presented from a DMDX platform (Forster and Forster, 2003), designed to precisely time the presentation of stimuli, sampled at a rate of 44100 Hz with 16–bit resolution. The ability to perceive stimuli were controlled for by a task where participants were asked to repeat 13 two-digit numbers directly after the presentation of each number. Prerecorded lists of 13 spoken two-digit numbers were then presented. After each list, the participants were asked to report particular list items, depending on the condition, updating or inhibition. Audio responses were recorded on an external tape recorder, as a basis for later scoring.

In the inhibition condition, participants were presented a list of value items spoken by a male or a female voice and asked to report at the end of the list the two odd value items spoken by the male voice. In the updating condition, participants were asked to report, the first item in the list as well as the two highest numbers in the list. There were three lists per condition and these were blocked. Before each block, the participants were provided with an oral instruction and whether to remember two numbers (inhibition) or three numbers (updating). In the updating condition the first item was, however, not counted as it is mainly an established way to increase memory load (Mishra et al., 2013a).

The EF score ranged from 0 to 2, six lists with one point for each correct recalled number of two possible within a list.

#### Verbal Ability and Communicative Skill

Verbal ability was evaluated by the vocabulary subtest in the Wechsler Adult Intelligence Scale (Wechsler, 1997). Participants were asked to define the meaning of words. Responses to items 1–33 were scored on the basis of the standardized scoring principles. A score of 2 was given for responses that demonstrated a good understanding of the word; a score of 1 was given for less elaborate correct responses and a score of 0 was given if the response was obviously wrong. The total score was computed by combining the scores for all items. The possible score was within the range 0–66.

Information about individual ability to use language and communicate was obtained from responses to the Alström Syndrome International questionnaire (Alström Syndrome International, 2010). The information was categorized in five levels, due to reported frequency of occurrence, from “never” to “almost always.” Information from the responses about ability to initiate and sustain communication, ability to pay attention to the topic, occurrence of repetitive use of language or words and odd rhythm of speech referring to a disorganization of temporal structures of the verbal stream (Zellner Keller and Keller, 2001), was specifically addressed.

### Procedure

Testing took place in a quiet room with normal acoustics. Hearing aids were adjusted and all participants used their fitted hearing aids. Audibility was ensured by adjusting hearing aid amplification and/or in some cases by the use of FM systems, where the participants chose the mode of amplification. Audibility was controlled for by a task where participants were to repeat numbers directly after the presentation of each number (Mishra et al., 2013b). The presentation level was adjusted to be comfortable for each individual participant and was held constant throughout testing, but the level was not verified. The tests of ToM, Phonological WM, EF, and Verbal ability were presented in the order mentioned. Assessments of hearing loss, measurements of visual acuity, visual field, and collection of questionnaire data were conducted separately. All testing in the study conform to regulatory standards.

### Data Analyses

Statistical Package for Social Sciences (SPSS) was used for statistical analyses. Independent *t*-tests were performed to examine group differences. Non-parametric, Mann–Whitney tests were used to examine differences between the both AS sub groups and between sub groups and the non-disabled control group. Spearmans’s rho non-parametric correlations were computed. A significance level of 0.05 was employed.

## Results

The results will be presented in three parts. First performance in ToM, phonological WM and EF are presented. Second, verbal ability and communicative skills are presented and related to EF and ToM. This is followed by a presentation of motor dysfunctions and sensory deficits in relation to EF and ToM.

### Theory-of-Mind Performance

A significant difference emerged between the AS group and the non-disabled control group with respect to the ability to produce correct mental inferences, where the AS group made significantly fewer correct mental state inferences than the control group (*t* = 5.062, df = 28, *p* < 0.0005).

### Phonological Working Memory Performance

A significant difference emerged between the AS group and the control group in phonological WM performance, where the AS group was outperformed by the non-disabled control group in proportion correct serially recalled non-words (*t* = 9.180, df = 28, *p* < 0.0005), in proportion correct recalled consonants (*t* = 11.323, df = 28, *p* < 0.0005) and in proportion correct recalled vowels and supra segmental accuracy (*t* = 14.869, df = 28, *p* < 0.0005).

A correlation was found in the AS group between audibility (ability to reproduce a two digit number directly after the presentation) and both reproduction of non-words (ρ = 718, *n* = 10, *p* = 0.019) and reproduction of consonants (ρ = 0.634, *n* = 10, *p* = 0.037), but no correlation was found between audibility and reproduction of vowels with total number of syllables.

### Executive Functioning Performance

A significant difference emerged between the AS group and the control group in EF, where the AS group performed at significantly lower levels than the control group in both inhibition (*t* = 4.105, df = 28, *p* < 0.0005) and updating (*t* = 4.603, df = 28, *p* < 0.0005). Individuals with AS that displayed an equal mental state understanding as the control group (*n* = 3) also showed equal performance with the control group in inhibition and updating, where two out three performed within I SD of the control group and the third marginally below. In contrast, individuals with AS that displayed significantly poorer mental state understanding than the control group (*n* = 7), also showed a significantly poorer performance in inhibition (*U* = 132.00, *N*_2_ = 27, *p* < 0.0005), and in updating (*U* = 5.559, *n* = 27, *p* = 0.002; **Table 2**).

**Table 2:** Percentage of correct mental inferences, serial recall of non-words correct consonants and correct vowels respectively, inhibition and updating (mean + SD) for non-disabled individuals (*n* = 20), individuals with AS (*n* = 10), individuals with AS and better ToM (*n* = 3), and individuals with AS and poorer ToM (*n* = 7).

	Non-disabled	AS	AS better	AS poorer	*p*
Corr. mental inferences	99.50 (1.34)	81.20 (14.89)			<0.01^∗∗^
Corr. mental inferences	99.50 (1.34)		100.00 (0.00)		ns
Corr. mental inferences	99.50 (1.34)			73.14 (8.97)	<0.01^∗∗^
SR correct consonants	76.15 (9.72)	29.36 (12.07)			<0.01^∗∗^
SR correct consonants	76.15 (9.72)		30.50 (10.89)		<0.01^∗∗^
SR correct consonants	76.15 (9.72)			26.33 (17.16)	<0.01^∗∗^
SR correct vowels	93.55 (1,23)	45.90 (3.56)			<0.01^∗∗^
SR correct vowels	93.55 (1,23)		47.50 (7.78)		<0.01^∗∗^
SR correct vowels	93.55 (1,23)			45.50 (12.38)	<0.01^∗∗^
Inhibition	94.10 (12.48)	59.09 (33.62)			<0.01^∗∗^
Inhibition	94.10 (12.48)		83.33 (16.50)		ns
Inhibition	94.10 (12.48)			50.00 (34.51)	<0.01^∗∗^
Updating	87.50 (14.08)	57.64 (21.56)			<0.01^∗∗^
Updating	87.50 (14.08)		72.33 (9.23)		ns
Updating	87.50 (14.08)			52.13 (22.63)	<0.01^∗∗^

*^∗∗^Difference is significant at p < 0.01*.

The degree of HI was not related to performance on inhibition or updating tasks in the AS group and did not correlate with inhibition nor with updating, Furthermore, no significant difference in audibility was displayed between the AS group and the control group. Correlations were, however, found in the AS group between production of correct mental inferences and both inhibition (ρ = 0.778, *n* = 10, *p* = 0.008) and updating (ρ = 0.740, *n* = 10, *p* = 0.014). In contrast, no correlations were obtained in the control group between production of correct mental inferences and inhibition or updating. Reported difficulties to pay attention to the topic in a dialog was negatively related to the production of correct mental inferences in AS (ρ = 0.740, *n* = 10, *p* = 0.014).

### Verbal Ability and Communicative Skills

A difference in verbal ability emerged between the AS group and the control group where the AS group performed at a significantly lower level (*t* = 3.552, df = 28, *p* = 0.001). A comparison between the group of AS individuals with a poorer mental state understanding and the control group displayed a significant difference in verbal ability, where the former group was outperformed (*U* = 123.00, *n* = 27, *p* = 0.003). Two out of three performed within I SD of the control group mean, No difference in verbal ability was in contrast displayed between AS individuals with a mental state understanding equal to the control group and the control group.

In the AS group verbal ability correlated with correct mental inferences (ρ = 0.737, *n* = 10, *p* = 0.015) and updating (ρ = 850, *n* = 10, *p* = 0.002), but not with inhibition, whereas in the control group verbal ability correlated neither with correct mental inferences, nor with updating or inhibition. Further, verbal ability was not related to reproduction of non-words, either in the AS group or in the control group. Verbal ability was related negatively to literal interpretations (ρ = -0.840, *n* = 9, *p* = 0.005) and repetitive use of language (ρ = -0.797, *n* = 9, *p* = 0.010) in the AS group.

The ability to initiate and sustain a conversation also correlated with correct mental inferences in the AS group (ρ = 0.753, *n* = 9, *p* = 0.019), with updating (ρ = 0.804, *n* = 804, *p* = 0.009) and with inhibition (ρ = 0.743, *n* = 9, *p* = 0.022). Ability to initiate and sustain communication correlated negatively with an odd rhythm of speech (ρ = -0.990, *n* = 8, *p* = 0.0005) in the AS group.

### Motor Dysfunctions and Sensory Deficits

In the AS group repetitive motor mannerisms as an infant or toddler, in contrast to any other motor dysfunction correlated negatively with correct mental inferences (ρ = -0.784, *n* = 8, *p* = 0.021), and inhibition (ρ = -784, *n* = 8, *p* = 0.021). Repetitive motor mannerisms in turn related to odd rhythm of speech (ρ = 986, *n* = 7, *p* < 0.0005).

There was no difference in occurrence of motor mannerisms between the two groups of AS individuals with, or without vestibular deficiencies, and no relation within the AS group between degree of visual impairment and motor mannerisms.

## Discussion

The purpose of this study was to explore how cognitive skills that are important for communication can account for differences in ToM performance among individuals with AS.

The AS group was outperformed by the non-disabled control group in ToM, phonological WM, EF, and verbal ability. Within the AS group, ToM was significantly predicted by EF and verbal ability but not by phonological WM. PTA_4_ significantly predicted phonological WM but not EF, verbal ability or ToM. The ability to infer meaning from incompletely received signals due to sensory loss, was related to updating capacity. The ability to initiate and sustain communication was related to both updating and inhibition of irrelevant information. A poor ability to initiate and sustain communication was related to a repetitive use of language and the occurrence of motor mannerisms. Individuals with AS that exhibited repetitive manners were accordingly characterized by a poorer inhibitory capacity.

The relation between ToM and verbal ability in AS is in agreement with earlier results for individuals with AS (Frölander et al., 2014) and also observed in other disabled populations (Hughes and Leekam, 2004; Fisher et al., 2005), as well as in populations of non-disabled individuals (Carlson et al., 1998; Ruffman et al., 2002). Advanced ToM required to capture the socially loaded ToM stories (Happe, 1994), is dependent on verbal understanding (Onishi et al., 2007). In addition, focused attention and cognitive efforts are required in advanced ToM processing (Fonagy and Luyten, 2009). These requirements are further increased by the challenge following a dual sensory loss (Dammeyer, 2010).

The progressive loss of hearing in AS primarily affects the high frequency range during the first decades (Marshall et al., 2007a) and is likely to hinder perception of consonants (Dubno et al., 2002; Pichora-Fuller and Souza, 2003). This may explain why the reproduction of consonants, but not of vowels that are of low frequency, correlated with audibility in the young adults with AS. Speech perception in ordinary communication improves with visual cues (Erber, 1969; Lidestam and Beskow, 2006; Zekveld et al., 2011), especially in challenging situations (Bernstein and Grant, 2009; Mishra et al., 2014). A progressive and early loss of vision in individuals with AS presumably negatively affects the ToM development as the visual loss in most cases are significant in a sensitive period for development, at the age of four. Visual loss is assumed to further limit the possibilities to perceive speech (Marshall et al., 2007a). The ability to understand speech under adverse conditions is, however, also dependent on cognitive skills such as WM capacity and executive functioning (Rönnberg et al., 2010).

In the present study, inhibition in the AS group was related to the ability to reproduce consonants in non-words. The reason for this outcome might be that the ability to inhibit irrelevant associations and thoughts affected the ability to correctly reproduce non-words (Miyake et al., 2000; Letho et al., 2003), The displayed differences in AS in inhibitory capacity is, however, of significant importance, as inhibition in addition was related to the ability to initiate and sustain communication in the AS group. This relation is reflecting that a prevention of irrelevant information from gaining attentional access is required in ongoing processes as communication (Lustig et al., 2007). Relations between inhibitory capacity and communication have been displayed in previous studies in other populations with disability as individuals with ASD (Ylvisaker and DeBonis, 2000; Noterdaeme et al., 2001; Mc Evoy et al., 2006), and Attention-Deficit/Hyperactivity Disorder (Ylvisaker and DeBonis, 2000). Mental references are developed through social interaction (Carpendale and Lewis, 2004), and the cognitive load of WM is, during normal conditions, relatively extensive (Slade and Ruffman, 2005). Challenges due to sensory loss would be expected to increase this load (Bernstein and Grant, 2009; Mishra et al., 2014). Thus, inhibitory capacity is important for individuals with AS in the development of ToM, as it may underpin the ability to initiate and sustain communication.

The ability to update WM with incoming information, and to compare this information with stored knowledge and infer meaning, is a process necessary, for example, to sentence comprehension (Rudner et al., 2011; Zekveld et al., 2011). In the present study, updating correlated with vocabulary in the AS group, which confirms a general relationship between updating and verbal ability (St Clair-Thompson and Gathercole, 2006). Poorer updating ability in the AS group was related to a preponderance of literal interpretations and a repetitive use of words. This suggests that vocabulary development may be dependent on updating in the AS group. In the present study, updating ability was further related to the production of correct mental inferences in the AS group. This may imply a mediating role for updating ability in vocabulary development, in turn promoting ToM.

Alström syndrome individuals with equal mental state understanding as the control group displayed a similar performance level as the control group in updating and in inhibition tasks. This is in line with previous results in non-disabled populations (Pernier and Lang, 1999; Carlson et al., 2004; Muller et al., 2012) as well as in other populations of individuals with disabilities, i.e., high functioning autism (Ozonoff et al., 1991) and cerebral palsy (Li et al., 2014). In the present study, among individuals with AS, poor ToM was related to reported problems in paying attention to the topic of a conversation. This pattern of results suggests that ToM in individuals with AS may be dependent on EF in general and inhibitory capacity in particular. Instead of the direct relation between ToM and EF observed in individuals with ASD (Zelazo and Carlson, 2012), we suggest a mediated relation (cf., Hughes and Leekam, 2004), where limitations in EF capacity impoverish communication and speech, already challenged in AS by hearing loss and additive severe visual loss, with negative consequences for ToM formation.

Odd rhythm of speech is part of a cluster of self-reported speech deficits in the AS group, related to poorer inhibitory capacity, communicative difficulties and to a lower frequency of produced correct mental inferences. Speech deficits were also associated with motor mannerisms in early childhood among individuals with AS in the present study. One possible explanation is that EF deficits in AS may cause speech mannerisms as well as early motor mannerisms, as both dysfunctions are related to motor control difficulties. These results are in line with similar findings for individuals with ASD (Livesey et al., 2006). In the present study, the ability to reproduce vowels in the AS group was also poor, despite the fact that perception of vowels did not seem to be affected by hearing loss. This indicates an inability to process information, which may also be related to EF. The findings in previous studies that inhibition of attention in general is strongly correlated with inhibition of action (Friedman and Miyake, 2004), further supports the notion that motor control problems in individuals with AS may be specifically related to poor inhibitory capacity.

Motor milestones are typically delayed in AS (Marshall et al., 2007a) as well as in other deafblind related syndromes such as Usher syndrome (Kimberling et al., 1989) and CHARGE syndrome (Dammeyer, 2012). Good balance is maintained by proprioception, vision and vestibular input. When vision and vestibular function are diminished, balance has to rely on proprioception which in many daily situations will give imbalance, unsteadiness and insecurity. Vestibular dysfunction is noted in many deafblind syndromes (Kimberling et al., 1989; Möller, 2003; Dammeyer, 2012), and frequent in AS (Marshall et al., 2007a). It causes motor delay (Marshall et al., 2007a), and might be one basis of the reported repetitive motor manners in the present study. However, AS individuals with vestibular dysfunction did not exhibit more motor mannerisms than AS individuals without vestibular dysfunction, suggesting dysfunctional top down processes instead (Diamond, 2013), defined as lack of capacity to perform deliberate kinds of processing (Rudner et al., 2008). Motor repetitive mannerisms are frequent in ASDs and related to lack of inhibitory control (Mahone et al., 2004; Mosconi et al., 2009), but also occur in non-spectrum disorders such as mental retardation (Bodfish et al., 2000). It is reasonable to also attribute such difficulties in AS to an inhibition deficit. Furthermore, repetitive mannerisms in AS were in contrast to other motor deviations, related to ToM. Similar findings have been made in ASD (Stoelb et al., 2004; Chamberlain et al., 2006), and this is probably reflecting the commonly displayed relation between ToM capacity and inhibition (Carlson and Moses, 2001).

Inhibitory control involves ability to control behavior (Diamond, 2013), and the prevalence of behavioral issues (i.e., obsessive compulsive disorder traits) in AS (Marshall et al., 2007a) probably reflects the poorer inhibitory capacity in many individuals. Lack of inhibitory control is associated with difficulty in regulating emotions (Carlson and Wang, 2007), exercising behavioral control, resisting temptation, and preventing impulses (Diamond, 2013) as well as the occurrence of repetitive behavior (Mosconi et al., 2009). Restrictions in inhibitory capacity that has been documented in a proportion of individuals with AS in this study could be the cause of observed behavioral issues that have been reported in this syndrome (Marshall et al., 2007a). Atypical behaviors are in general cross situational (Funder and Colvin, 1991), and biological influence specifically on behavioral inhibition has been confirmed (Matheny, 1989).

Neurocognitive impairments are frequently demonstrated in individuals with ciliary dysfunction (Lee and Gleeson, 2010). Brain abnormalities have also been established in individuals with AS, mainly in posterior regions (Citton et al., 2013; Manara et al., 2015). AS has a significant phenotypic overlap with Bardet Biedl syndrome (BBS). Motor mannerisms are displayed in both syndromes (Dyer et al., 1994; Marshall et al., 2007b). Such mannerisms have, however, also been observed in non-ciliopathy disorders, i.e., in individuals with visual impairment of varying etiology; (Fazzi et al., 1999; Molloy and Rowe, 2011). However, no relationship was seen between motor mannerisms of individuals with AS in the present study and degree of visual impairment, suggesting that the main cause of mannerisms may not be sensory loss. In Joubert syndrome, another ciliopathy where sensory dysfunction is not as frequently displayed as in AS and BBS, repetitive and stereotyped motor mannerisms have, however, been reported, and related to EF deficits caused by abnormalities in cerebellum (Steinlin, 2007). Deficits in cerebellar areas are known to relate to deficits in EF (Cardoso et al., 2014) and individuals with higher EF have been shown to have greater recruitment of cerebellar regions within a fronto-parietal resting state network (Reineberg et al., 2015). Cerebellar anomalies have in fact been observed in individuals with AS (Yilmaz et al., 2006), but no consistent findings in these areas have been discovered in the AS population (Citton et al., 2013). Thus, individual cerebellar anomalies may account for some of the EF deficit found in individuals with AS in the present study. In contrast, in previous studies of, i.e., deaf and hard of hearing individuals, poorer EF performance has been recognized as a secondary consequence of sensory loss (Rhine-Kahlback, 2004). No significant relation between degree of sensory loss and EF performance was, however, displayed in the present study. Aside of brain anomalies, this suggests potential external factors of importance for development of EF capacity in AS, illustrated by training effects in both non-disabled children (Thorell et al., 2009) and the elderly (Nagamatsu et al., 2012).

Autism spectrum traits have been observed in some individuals with AS (Marshall et al., 2007a), and in the present study the AS group displayed a significantly poorer ToM than the group of non-disabled controls. A general decrease in ToM capacity is typical for individuals with ASD (Baron-Cohen, 1989). The results from the present study, however, indicates a varied performance level in ToM in the AS group, that is highly dependent on verbal ability, whereas the threshold for language impact in ASD is markedly high (Hughes and Leekam, 2004).

Theory-of-mind capacity is related to EFs in disabled as well as non-disabled populations, where the direct role of inhibition in numerous studies have been stressed in development of an understanding that another person may hold a belief different from one self (Carlson and Moses, 2001). The within group variance in ToM in the present study, rather indicates an indirect role of EF in ToM development in AS, mediated by communicative skills and verbal ability. A better ability to inhibit noise and to update information might compensate for the consequences of sensory loss in communication. An additive factor of importance to understand difficulties in individuals with AS to develop advanced ToM is the significant loss of vision already at the age of four, which is a sensitive age in development of ToM. In a previous study early onset of visual loss in AS was accordingly found to relate to poorer ToM capacity (Frölander et al., 2014). Early visual loss in addition to progressing hearing loss over time constitute a challenge to communication. Variance in executive functioning could, however, explain a significant part of the variance in ToM in AS, pointing at an indirect role of EF in ToM development, in contrast to the direct relation between EF and ToM displayed in ASD.

This study of cognitive prerequisites for ToM development is complicated due to the small population, reflecting the low prevalence of AS. Moreover the present population displayed a high degree of heterogeneity in performance and ceiling effects were recognized in the non-disabled control group. A division of the population into subgroups made between and within group comparisons possible, and also uncovered patterns of interaction. The influence of cognitive skills in ToM performance is, however, only for certain when audibility is fully restored (Akeroyd, 2008). This was accomplished in the EF test but not in the test of phonological WM. It is possible that the differences in presentation level between participants influenced the pattern of correlation. However, there is no reason to believe that presentation level systematically covaried with either of the variables of interest. Thus that any incidental differences in presentation level are likely to have weakened rather than strengthened the association. By relating performance on the phonological WM test, to individual performance on the EF test an investigation of the compensatory role of EF, when the perception of speech signals is challenged, was, however, accomplished.

## Conclusion

In the present study the complication of hearing and visual loss in individuals with AS is addressed and related to development of ToM. The results confirm previous results showing that ToM is related to verbal ability in individuals with AS. A novel finding is that the results imply a compensatory role of EF in ToM development in AS. We suggest that this relation reflects the challenge of dual sensory loss and the importance of EF in developing the ability to perceive and process input from the social environment. It is likely that good updating capacity in individuals with AS enables inferences of meaning from incompletely received signals. Updating capacity contributes to verbal ability that is of importance for ToM development. Further, good inhibitory capacity enables sustained social interaction, in spite of the challenging conditions during communication. Poorer inhibitory capacity could be one cause of the observed mannerisms in individuals with AS, which may further decrease their opportunities to participate in communication.

Recommendations; The conclusions of the present study imply that rehabilitation of individuals with AS should focus on development of verbal ability and EF. Cognitive intervention that focuses on EF may be effective in order to support development of ToM as a basis for enhanced social participation and reduction of abnormal behavior. The apparent association between ToM and EFs in AS, however, needs to be further elaborated and replicated. As the mutations causing AS is actively expressed in most organs brain anomalies have been reported in some individuals with AS (Marshall et al., 2007a; Citton et al., 2013; Manara et al., 2015), further neurological studies need to be conducted. In addition, implementation of cognitive methods to improve verbal ability and EFs in individuals with AS would be of interest in future studies.

## Conflict of Interest Statement

The authors declare that the research was conducted in the absence of any commercial or financial relationships that could be construed as a potential conflict of interest.

## References

[B1] Akeroyd M. A. (2008). Are individual differences in speech reception related to individual differences in cognitive ability? A survey of twenty experimental studies with normal and hearing impaired adults. *Int. J. Audiol.* 47 S53–S71. 10.1080/1499202802301142 19012113

[B2] Alström Syndrome International. (2010). *Alström Syndrome Questionnaire*. Mount Deser, ME: Alström Syndrome International.

[B3] Baddeley A. (2012). Working memory: theories, models, and controversies. *Annu. Rev. Psychol.* 63 1–29. 10.1146/annurev-psych-120710-100422 21961947

[B4] Baron-Cohen S. (1989). Are autistic children “behaviorists”? An examination of their mental-physical and appearance-reality distinctions. *J. Autism Dev. Disord.* 19 579–600. 2606886 10.1007/BF02212859

[B5] Belmonte M. K. Bourgeron T. (2006). Fragile X syndrome and autism at the intersection of genetic and neural networks. *Nat. Neurosci.* 9 1221–1225. 10.1038/nn1765 17001341

[B6] Bernstein J. G. W. Grant K. W. (2009). Auditory and auditory-visual intelligibility of speech in fluctuating maskers for normal-hearing and hearing-impaired listeners. *J. Acoust. Soc. Am.* 125 3358–3372. 10.1121/1.3110132 19425676

[B7] Bishop D. Adams C. (1989). Conversational characteristics of children with semantic-pragmatice disorder: II What features lead to a judgement of innappropriacy? *Int. J. Lang. Commun. Disord.* 24 241–263. 10.3109/13682828909019890 2627545

[B8] Bodfish J. W. Symons F. J. Parker D. E. Lewis M. H. (2000). Varieties of repetitive behavior in autism: comparison to mental retardation. *J. Autism Dev. Disord.* 30 237–243. 10.1023/A:1005596502855 11055459

[B9] Burgess P. W. Simons J. S. (eds) (2005). *Theories of Frontal Lobe Executive Function: Clinical Applications.* Oxford: Oxford University Press.

[B10] Cardoso C. D. Branco L. D. Cotrena C. Kristensen C. H. Bakos D. D. S. Fonseca R. P. (2014). The impact of frontal and cerebellar lesions on decision making: evidence from the Iowa Gambling Task. *Front. Neurosci.* 8:61 10.3389/fnins.2014-00061 PMC3986592 24782697

[B11] Carlson S. M. Moses L. J. (2001). Individual differences in inhibitory control and children’s theory of mind. *Child Dev.* 72 1032–1053. 10.1111/1467-8624.00333 11480933

[B12] Carlson S. M. Moses L. J. Breton C. (2002). How specific is the relation between executive function and theory of mind? Contributions of inhibitory control and working memory. *Infant Child Dev.* 11 73–92. 10.1002/icd.298

[B13] Carlson S. M. Moses L. J. Claxton L. J. (2004). Individual differences in executive functioning and theory of mind: an investigation of inhibitory control and planning ability. *J. Exp. Child Psychol.* 87 299–319. 10.1016/j.jecp.2004.01.002 15050456

[B14] Carlson S. M. Moses L. J. Hix H. R. (1998). The role of inhibitory processes in young children’s difficulties with deception and false belief. *Child Dev.* 69 672–691. 10.1111/j.1467-8624.1998.00672.x 9680679

[B15] Carlson S. M. Wang T. S. (2007). Inhibitory control and emotion regulation in preschool children. *Cogn. Dev.* 22 489–510. 10.1016/j.cogdev.2007.08.002

[B16] Carpendale J. I. M. Lewis C. (2004). Constructing an understanding of mind: the development of children’s social understanding within social interaction. *Behav. Brain Sci.* 27 79–96. 10.1017/S0140525X04000032 15481944

[B17] Chamberlain S. R. Fineberg N. A. Blackwell A. D. Robbins T. W. Sahakian B. J. (2006). Motor inhibition and cognitive flexibility in obsessive-compulsive disorder and trichotillomania. *Am. J. Psychiatry* 163 1282–1284. 10.1176/appi.ajp.163.7.1282 16816237

[B18] Citton V. Favaro A. Bettini V. Gabrieli J. Milan G. Greggio N. A. (2013). Brain involvement in Alström syndrome. *Orphanet J. Rare Dis.* 8 24 10.1186/1750-1172-8-24 PMC3584911 23406482

[B19] Cornish K. Burack J. A. Rahman A. Munir F. Russo N. Grant C. (2005). Theory of mind deficits in children with fragile X syndrome. *J. Intellect. Disabil. Res.* 49 372–378. 10.1111/j.1365-2788.2005.00678.x 15817054

[B20] Dammeyer J. (2010). Interaction of dual sensory loss, cognitive function, and communication in people who are congenitally deaf-blind. *J. Vis. Impair. Blind.* 104 719–725.

[B21] Dammeyer J. (2012). Development and characteristics of children with Usher syndrome and CHARGE syndrome. *Int. J. Pediatr. Otorhinolaryngol.* 76 1292–1296. 10.1016/j.ijporl.2012.05.021 22721527

[B22] Davis H. L. Pratt C. (1995). The development of children’s theory of mind: the working memory explanation. *Aust. J. Psychol.* 47 25–31. 10.1080/00049539508258765

[B23] Diamond A. (2013). Executive functions. *Annu. Rev. Psychol.* 64 135–168. 10.1146/annurev-psych-113011-143750 23020641 PMC4084861

[B24] Dubno J. R. Ahlstrom J. B. Hoewitz A. R. (2002). Spectral contributions to the benefit from spatial separation of speech and noise. *J. Speech Lang. Hear. Res.* 45 1297–1310. 10.1044/1092-4388(2002/104) 12546495

[B25] Dyer D. S. Wilson M. E. Small K. W. Pai G. S. (1994). Alstrom syndrome: a case misdiagnosed as Bardet-Biedl syndrome. *J. Pediatr. Ophthalmol. Strabismus* 31 272–274. 7807310 10.3928/0191-3913-19940701-19

[B26] Erber N. P. (1969). Interaction of audition and vision in the recognition of oral speech stimuli. *J. Speech Lang. Hear. Res.* 12 423–425. 10.1044/jshr.1202.423 5808871

[B27] Espy K. A. Molfese D. L. Molfese V. J. Modglin A. (2004). Development of auditory event-related potentials in young children and relations to word-level reading abilities at age 8 years. *Ann. Dyslexia* 54 9–38. 10.1007/s11881-004-0002-3 15765002 PMC2729145

[B28] Fazzi E. Lanners J. Danova S. Ferrarri-Ginevra O. Gheza C. Luparia A. (1999). Stereotyped behaviours in blind children. *Brain Dev.* 21 522–528. 10.1016/S0387-7604(99)00059-5 10598052

[B29] Fine C. Lumsden J. Blair R. J. (2001). Dissociation between ‘theory of mind’ and executive functions in a patient with early left amygdala damage. *Brain* 124 287–298. 10.1093/brain/124.2.287 11157556

[B30] Fisher N. Happé F. Dunn J. (2005). The relationship between vocabulary, grammar, and false belief task performance in children with autistic spectrum disorders and children with moderate learning difficulties. *J. Child Psychol. Psychiatry* 46 409–419. 10.1111/j.1469-7610.2004.00371.x 15819650

[B31] Fletcher P. C. Happé F. Frith U. Baker S. C. Dolan R. J. Frackowiak R. S. (1995). Other minds in the brain: a functional imaging study og “theory of mind” in story comprehension. *Cognition* 57 109–128. 10.1016/0010-0277(95)00692-R 8556839

[B32] Fonagy P. Luyten P. (2009). A developmental, mentalization-based approach to the understanding and treatment of borderline personality disorder. *Dev. Psychopathol.* 21 1355–1381. 10.1017/S0954579409990198 19825272

[B33] Forster K. I. Forster J. C. (2003). DMDX: a Windows display program with millisecond accuracy. *Behav. Res. Methods Instrum. Comput.* 35 116–124. 10.3758/BF03195503 12723786

[B34] Friedman N. P. Miyake A. (2004). The relations among inhibition and interference control functions: a latent-variable analyses. *J. Exp. Psychol.* 133 101–135. 10.1037/0096-3445.133.1.101 14979754

[B35] Frölander H. E. Moller C. Marshall J. D. Sundqvist A. Ronnasen B. Falkensson L. (2014). Theory-of-mind in adolescents and young adults with Alstrom syndrome. *Int. J. Pediatr. Otorhinolaryngol.* 78 530–536. 10.1016/j.ijporl.2013.12.038 24485176

[B36] Funder D. C. Colvin C. R. (1991). Explorations in behavioral consistency: properties of persons, situations and behaviors. *J. Pers. Soc. Psychol.* 60 773–794. 10.1037/0022-3514.60.5.773 2072255

[B37] Gathercole S. E. Pickering S. J. (2000). Assessment of working memory in six – and seven-year-old children. *J. Educ. Psychol.* 92 377–390. 10.1037/0022-0663.92.2.377

[B38] Grover S. Fishman G. A. Anderson R. J. Alexander K. R. Derlacki D. J. (1997). Rate of visual field loss in retinitis pigmentosa. *Ophthalmology* 104 460–465. 10.1016/S0161-6420(97)30291-7 9082273

[B39] Happe F. G. (1994). An advanced test of theory of mind: understanding of story characters’ thoughts and feelings by able autistic, mentally handicapped, and normal children and adults. *J. Autism Dev. Disord.* 24 129–154. 10.1007/BF02172093 8040158

[B40] Happe F. Ehlers S. Fletcher P. Frith U. Johansson M. Gillberg C. (1996). ‘Theory of mind’ in the brain. Evidence from a PET scan study of Asperger syndrome. *Neuroreport* 8 197–201. 9051780 10.1097/00001756-199612200-00040

[B41] Hartshorne T. S. Hefner M. A. Davenport S. L. (2005). Behavior in CHARGE syndrome: introduction to the special topic. *Am. J. Med. Genet. A* 133A 228–231. 10.1002/ajmg.a.30541 15637707

[B42] Hughes C. (1998a). Executive function in preschoolers: links with theory of mind and verbal ability. *Br. J. Dev. Psychol.* 16 233–253. 10.1111/j.2044-835X.1998.tb00921.x

[B43] Hughes C. (1998b). Finding your marbles. does preschooler’s strategic behaviour predict later understanding of mind? *Dev. Psychol.* 34 1326–1339. 10.1037/0012-1649.34.6.1326 9823515

[B44] Hughes C. Leekam S. (2004). What are the links between theory of mind and social relations? Review, reflections and new directions for studies of typical and atypical development. *Soc. Dev.* 13 590–619. 10.1111/j.1467-9507.2004.00285.x

[B45] Jolliffe T. Baron-Cohen S. (1999). The strange stories test: a replication with high-functioning adults with Autism or Asperger syndrome. *J. Autism Dev. Disord.* 29 395–406. 10.1023/A:1023082928366 10587886

[B46] Joseph R. M. Tager-Flusberg H. (2004). The relationship of theory of mind and executive functions to symptom type and severity in children with autism. *Dev. Psychopathol.* 16 137–155. 10.1017/S095457940404444X 15115068 PMC1201455

[B47] Keenan T. (1998). Memory span as apredictor of false belief understanding. *NZ. J. Psychol.* 27 36–43.

[B48] Keenan T. Olson D. R. Marini Z. (1998). Working memory and childrens developing understanding of mind. *Aust. J. Psychol.* 50 76–82. 10.1080/00049539808257537

[B49] Kimberling W. J. Möller C. Davenport S. L. Lund G. Grissom T. J. Priluck I. (1989). Usher syndrome: clinical findings and gene localization studies. *Laryngoscope* 99 66–72. 10.1288/00005537-198901000-00013 2562904

[B50] Landa R. J. Goldberg M. C. (2005). Language, social, and executive functions in high functioning autism: a continuum of performance. *J. Autism Dev. Disord.* 35 557–573. 10.1007/s10803-005-0001-1 16211332

[B51] Lee J. H. Gleeson J. G. (2010). The role of primary cilia in neuronal function. *Neurobiol. Dis.* 38 167–172. 10.1016/j.nbd.2009.12.022 20097287 PMC2953617

[B52] Leslie A. M. Friedman O. German T. P. (2004). Core mechanisms in “theory of mind.” *Trends Cogn. Sci.* 8 528–533. 10.1016/j.tics.2004.10.001 15556021

[B53] Letho J. E. Juujärvi P. Kooistra L. Pulkkinen L. (2003). Dimensions of executive functioning: evidence from children. *Br. J. Dev. Psychol.* 21 59–80. 10.1348/026151003321164627

[B54] Li G. Vega R. Nelms K. Gekakis N. Goodnow C. Mcnamara P. (2007). A role for Alstrom syndrome protein, alms1, in kidney ciliogenesis and cellular quiescence. *PLoS Genet.* 3:e8 10.1371/journal.pgen.0030008 PMC1761047 17206865

[B55] Li X. Wang K. Wu J. Hong Y. Zhao J. Feng X. (2014). The link between impaired theory of mind and executive function in children with cerebral palsy. *Res. Dev. Disabil.* 35 1686–1693. 10.1016/j.ridd.2014.03.017 24685096

[B56] Lidestam B. Beskow J. (2006). Visual phonemic ambiguity and speechreading. *J. Speech Lang. Hear. Res.* 49 835–847. 10.1044/1092-4388(2006/059) 16908878

[B57] Livesey D. Keen J. Rouse J. White F. (2006). The relationship between measures of executive function, motor performance and externalising behaviour in 5- and 6-year-old children. *Hum. Mov. Sci.* 25 50–64. 10.1016/j.humov.2005.10.008 16442172

[B58] Lustig C. Hasher L. Zacks R. T. (2007). “Inhibitory deficit theory: recent developments in a “New View”,” in *Inhibition in Cognition*, eds Gorfein D. S. Macleod C. M. (Washington, DC: American Psychological Association) 145–162.

[B59] Lyxell B. Wass M. Sahlén B. Samuelson C. Asker Árnason L. Ibertsson T. (2009). Cognitive development, reading and prosodic skills in children with cochlear implants. *Scand. J. Psychol.* 50 463–474. 10.1111/j.1467-9450.2009.00754.X 19778394

[B60] Mahone M. Bridges D. Prahme C. Singer H. S. (2004). Repetitive arm and hand movements (complex motor stereotypies) in children. *J. Pediatr.* 146 391–395. 10.1016/j.jpeds2004.06.014 15343197

[B61] Manara R. Citton V. Maffei P. Marshall J. D. Naggert J. K. Milan G. (2015). Degeneration and plasticity of the optic pathway in Alström syndrome. *Am. J. Neuroradiol.* 36 160–165. 10.317/ajnr.A4115 25355816 PMC7965932

[B62] Marshall J. D. Beck S. Maffei P. Naggert J. K. (2007a). Alstrom syndrome. *Eur. J. Hum. Genet.* 15 1193–1202. 10.1038/sj.ejhg.5201933 17940554

[B63] Marshall J. D. Hinman E. G. Collin G. B. Beck S. Cerqueira R. Maffei P. (2007b). Spectrum of ALMS1 variants and evaluation of genotype-phenotype correlations in Alstrom syndrome. *Hum. Mutat.* 28 1114–1123. 10.1002/humu.20577 17594715

[B64] Marshall J. D. Maffei P. Collin G. B. Naggert J. K. (2011). Alström syndrome: genetics and clinical Overview. *Curr. Genomics* 12 225–235. 10.2174/13892021175677912 22043170 PMC3137007

[B65] Matheny A. P. Jr. (1989). Children’s behavioral inhibition over age and across situations: genetic similarity for a trait during change. *J. Pers.* 57 215–235. 10.1111/j.1467-6494.1989.tb0048.x 2769555

[B66] Mc Evoy R. E. Rogers S. J. Pennington B. F. (2006). Executive function and social communication deficits in young Autistic children. *J. Child Psychol. Psychiatry* 34 563–578. 10.1111/j.1469-7610.1993.tb01036.x 7685360

[B67] Minter M. Hobson R. P. Bishop M. (1998). Congenital visual impairment and ‘theory od mind’. *Br. J. Dev. Psychol.* 16 183–196. 10.1111/j.2044-835X.1998.tb00918.x

[B68] Mishra S. Lunner T. Stenfelt S. Ronnberg J. Rudner M. (2013a). Seeing the talker’s face supports executive processing of speech in steady state noise. *Front. Syst. Neurosci.* 7:96 10.3389/fnsys.2013.00096 PMC3840300 24324411

[B69] Mishra S. Lunner T. Stenfelt S. Ronnberg J. Rudner M. (2013b). Visual information can hinder working memory processing of speech. *J. Speech Lang. Hear. Res.* 56 1120–1132. 10.1044/1092-4388(2012/12-0033) 23785180

[B70] Mishra S. Stenfelt S. Lunner T. Rönnberg J. Rudner M. (2014). Cognitive spare capacity in older adults with hearing loss. *Front. Aging Neurosci.* 6:96 10.3389/fnaqi.2014.00096 PMC4033040 24904409

[B71] Mitchell P. Riggs K. J. (2000). *Children’s Reasoning and the Mind.* Hove: Psychology Press.

[B72] Miyake A. Naomi P. Friedman N. P. Emerson M. J. Witzki A. H. Howerter A. (2000). The unity and diversity of executive functions and their contributions to complex “frontal lobe” tasks: a latent variable analysis. *Cogn. Psychol.* 4 49–100. 10.1006/cogp.1999.0734 10945922

[B73] Möller C. (2003). Deafblindness: living with sensory deprivation. *Lancet* 362 46–47. 10.1016/S0140-673(03)15074-X 14698128

[B74] Möller C. (2007). “Deafblindness,” in *Genes Hearing and Deafness*, eds Martini A. Stephens D. Read A. (Oxford: Informa healthcare) 55–61.

[B75] Molloy A. Rowe F. J. (2011). Manneristic behaviors of visually impaired children. *Strabismus* 19 77–84. 10.3109/09273972.2011.600417 21870909

[B76] Mosconi M. W. Kay M. Cruz A.-M. Seidenfeld A. Guter S. Stanford L. D. (2009). Impaired inhibitory control is associated with higher-order repetitive behaviors in autism spectrum disorders. *Psychol. Med.* 39 1559–1566. 10.1017/S0033291708004984 19154646 PMC3145414

[B77] Muller U. Liebermann-Finestone D. P. Carpendale J. I. M. Hammond S. I. Bibok M. B. (2012). Knowing minds, controlling actions: the developmental relations between theory of mind and executive function from 2 – 4 years of age. *J. Exp. Child Psychol.* 111 331–348. 10.1016/j.jecp.2011.08.014 22024385

[B78] Nagamatsu L. S. Handy T. C. Hsu C. L. Voss M. Liu-Ambrose T. (2012). Resistance training promotes cognitive and functional brain plasticity in seniors with probable mild cognitive impairment. *Arch. Intern. Med.* 172 666–668. 10.1001/archinternmed.2012.379 22529236 PMC3514552

[B79] Noterdaeme M. Amorosa H. Milldenberger K. Sitter S. Minow F. (2001). Evaluation of attention problems in children with autism or children with specific language disorder. *Eur. Child Adolesc. Psychiatry* 10 58–66. 10.1007/s007870170048 11315537

[B80] Onishi K. H. Baillargeon R. Leslie A. M. (2007). 15-month-old infants detect violations in pretend scenarios. *Acta Psychol. (Amst.)* 124 106–128. 10.1016/j.actpsy.2006.09.009 17107649 PMC3351386

[B81] Ozonoff S. Jensen J. (1999). Brief report: specific executive function profiles in three neurodevelopmental disorders. *J. Autism Dev. Disord.* 29 171–177. 10.1023/A:1023052913110 10382139

[B82] Ozonoff S. Pennington B. F. Rogers S. J. (1991). Executive function deficits in high-functioning autistic individuals: relationship to theory of mind. *J. Child Psychol. Psychiatry* 32 1081–1105. 10.1111/j.1469-7610.1991.tb00351.x 1787138

[B83] Perner J. Lang B. (1999). Development of theory of mind and executive control. *Trends Cogn. Sci.* 3 337–344. 10.1016/S1364-6613(99)01362-5 10461196

[B84] Pernier J. Lang B. (1999). Development of theory-of-mind and executive control. *Trends Cogn. Sci.* 3 337–344. 10.1016/S1364-6613(99)01362-5 10461196

[B85] Peterson C. C. (2004). Theory-of-mind development in oral deaf children with cochlear implants or conventional hearing aids. *J. Child Psychol. Psychiatry* 45 1096–1106. 10.1111/j.1469-7610.2004.t01-1-00302.x 15257666

[B86] Pichora-Fuller M. K. Souza P. E. (2003). Effects of aging on auditory processing of speech. *Int. J. Audiol.* 42 11–16. 10.3109/14992020309074638 12918623

[B87] Premack D. Woodruff G. (1978). Does chimpanzee have atheory of mind. *Behav. Brain Sci.* 4 515–526. 10.1017/S0140525X00076512

[B88] Reineberg A. E. Andrews-Hanna J. R. Depue B. E. Friedman N. P. Banich M. T. (2015). Resting-state networks predict individual differences in common and specific aspects of executive function. *Neuroimage* 104 69–78. 10.1016/j.neuroimage.2014.09.045 25281800 PMC4262251

[B89] Rhine-Kahlback S. (2004). *Language Differences, Executive Functioning, and Social Skills in Deaf Children.* Washington, DC: Gallaudet University.

[B90] Roch-Levecq A.-C. (2006). Production of basic emotions by children with congenital blindness: evidence for the embodiment of theory of mind. *Br. J. Dev. Psychol.* 24 507–528. 10.1348/026151005X50663

[B91] Rönnberg J. Rudner M. Lunner T. Zekveld A. A. (2010). When cognition kicks in: working memory and speech understanding in noise. *Noise Health* 12 263–269. 10.4103/1463-1741.70505 20871181

[B92] Rowe A. D. Bullock P. R. Polkey C. E. Morris R. G. (2001). ‘Theory of mind’ impairment and their relationship to executive functioning following frontal lobe excisions. *Brain* 124 600–616. 10.1093/brain/124.3.600 11222459

[B93] Rudner M. Foo C. Sundewall-Thorén E. Lunner T. Rönnberg J. (2008). Phonological mismatch and explicit cognitive processing in a sample of 102 hearing-aid users. *Int. J. Audiol.* 47 S91–S98. 10.1080/14992020802304393 19012116

[B94] Rudner M. Rönnberg J. Lunner T. (2011). Working memory supports listening in noise for persons with hearing impairment. *J. Am. Acad. Audiol.* 22 156–167. 10.3766/jaaa.22.3.4 21545768

[B95] Ruffman T. Slade L. Crowe E. (2002). The relation between children’s and mothers’ mental state language and theory-of-mind understanding. *Child Dev.* 73 734–751. 10.1111/1467-8624.00435 12038548

[B96] Sabbagh M. A. Hopkins S. F. Benson J. E. Flanagan J. R. (2010). Conceptual change and preschoolers’ theory of mind: evidence from load-force adaptation. *Neural Netw.* 23 1043–1050. 10.1016/j.neunet.2010.08.007 20851572

[B97] Slade L. Ruffman T. (2005). How language does (and does not) relate to theory of mind: a longitudinal study of syntax, semantics, working memory and false belief. *Br. J. Dev. Psychol.* 23 117–141. 10.1348/026151004X21332

[B98] St Clair-Thompson H. L. Gathercole S. E. (2006). Executive functions and achievements in school: shifting, updating, inhibition, and working memory. *Q. J. Exp. Psychol. (Hove)* 59 745–759. 10.1080/17470210500162854 16707360

[B99] Steinlin M. (2007). The cerebellum in cognitive processes: supporting studies in children. *Cerebellum* 6 237–241. 10.1080/14734220701344507 17786820

[B100] Stoelb M. Yarnal R. Miles J. Takahashi N. Farmer J. E. Mccathren R. (2004). Children with autism a retrospective study of the importance of physical dysmorphology. *Focus Autism Other Dev. Disabl.* 19 66–77. 10.1177/10883576040190020101

[B101] Sundqvist A. Lyxell B. Jonsson R. Heimann M. (2014). Understanding minds: early cochlear implantation and the development of theory of mind in children with profound hearing impairment. *Int. J. Pediatr. Otorhinolaryngol.* 78 537–543. 10.1016/j.ijporl.2013.12.039 24485174

[B102] Thorell L. B. Lindqvist S. Bergman Nutley S. Bohlin G. Klingberg T. (2009). Training and transfer effects of executive functions in preschool children. *Dev. Sci.* 12 106–113. 10.1111/j.1467-7687.2008.00745.x 19120418

[B103] Wechsler D. (1997). *Wechsler Adult Intelligence Scale: Administration and Scoring Manual.* San Antonio, TX: The Psychological Corporation.

[B104] Wellman H. M. Cross D. Watson J. (2001). Meta-analysis of theory-of-mind development: the truth about false belief. *Child Dev.* 72 655–684. 10.1111/1467-8624.00304 11405571

[B105] Wellman H. M. Woolley J. D. (1990). From simple desires to ordinary beliefs: the early development of everyday psychology. *Cognition* 35 245–275. 10.1016/0010-0277(90)90024-E 2364653

[B106] White S. Hill E. Happe F. Frith U. (2009). Revisiting the strange stories: revealing mentalizing impairments in autism. *Child Dev.* 80 1097–1117. 10.1111/j.1467-8624.2009.01319.x 19630896

[B107] Yilmaz C. Çaksen H. Yilmaz N. Güven A. S. Arslan D. Cesur Y. (2006). Alström syndrome associated with cerebral involvement: an unusual presentation. *Eur. J. Gen. Med.* 3 32–34.

[B108] Ylvisaker M. DeBonis D. (2000). Executive function impairment in adolescence: TBI and ADHD. *Top. Lang. Disord.* 20 29–57. 10.1097/00011363-200020020-00005

[B109] Zekveld A. A. Rudner M. Johnsrude I. S. Festen J. M. Van Beck H. M. Rönnberg J. (2011). The influence of semantically related and unrelated text cues on intelligibility of sentences in noise. *Ear Hear.* 32 16–25. 10.1097/AUD.0b013e318228036a 21826004

[B110] Zelazo P. D. Burack J. A. Benedetto E. Frye D. (1996). Theory of mind and rule use in individuals with Down’s syndrome: a test of the uniqueness and specificity claims. *J. Child Psychol. Psychiatry* 37 479–484. 10.1111/j.1469-7610.1996.tb01429.x 8735448

[B111] Zelazo P. D. Carlson S. M. (2012). Hot and cool executive function in childhood and adolescence: development and plasticity. *Child Dev. Perspect.* 6 354–360. 10.1111/j.1750-8606.2012.00246.x

[B112] Zellner Keller B. Keller E. (eds) (2001). *A Non-Linear Rhythmic Component in Various Styles of Speech.* Chichester: John Wiley.

